# Environmental enrichment effects after early stress on behavior and functional brain networks in adult rats

**DOI:** 10.1371/journal.pone.0226377

**Published:** 2019-12-12

**Authors:** Héctor González-Pardo, Jorge L. Arias, Guillermo Vallejo, Nélida M. Conejo

**Affiliations:** 1 Laboratory of Neuroscience, Department of Psychology and Institute of Neuroscience of the Principality of Asturias (INEUROPA), University of Oviedo, Oviedo, Spain; 2 Methodology Area, Department of Psychology and Institute of Neuroscience of the Principality of Asturias (INEUROPA), University of Oviedo, Oviedo, Spain; Technion Israel Institute of Technology, ISRAEL

## Abstract

Early life stress is associated with long-term and pervasive adverse effects on neuroendocrine development, affecting normal cognitive and emotional development. Experimental manipulations like environmental enrichment (EE) may potentially reverse the effects of early life stress induced by maternal separation (MS) paradigm in rodents. However, the functional brain networks involved in the effects of EE after prolonged exposure to MS have not yet been investigated. In order to evaluate possible changes in brain functional connectivity induced by EE after MS, quantitative cytochrome c oxidase (CCO) histochemistry was applied to determine regional brain oxidative metabolism in adult male rats. Unexpectedly, results show that prolonged MS during the entire weaning period did not cause any detrimental effects on spatial learning and memory, including depressive-like behavior evaluated in the forced-swim test, and decreased anxiety-like behavior. However, EE seemed to alter anxiety- and depression-like behaviors in both control and MS groups, but improved spatial memory in the latter groups. Analysis of brain CCO activity showed significantly lower metabolic capacity in most brain regions selected in EE groups probably associated with chronic stress, but no effects of MS on brain metabolic capacity. In addition, principal component analysis of CCO activity revealed increased large-scale functional brain connectivity comprising at least three main networks affected by EE in both MS and control groups. Moreover, EE induced a pattern of functional brain connectivity associated with stress and anxiety-like behavior as compared with non-enriched groups. In conclusion, EE had differential effects on cognition and emotional behavior irrespective of exposure to MS. In view of the remarkable effects of EE on brain function and behavior, implementation of rodent housing conditions should be optimized by evaluating the balance between scientific validity and animal welfare.

## Introduction

It is widely acknowledged that exposure to social stress during the early postnatal period is linked to averse long-term outcomes on brain development and adult behavior. In particular, early life stress significantly increases the risk of developing mental disorders in adulthood [1,2]. Both childhood physical abuse and neglect are critical environmental risk factors linked to the development of several mental disorders like major depression, bipolar disorder, anxiety disorders, posttraumatic stress disorder, substance use disorders, and even schizophrenia [3]. Animal models of early life stress in primates and rodents usually rely on the disruption of mother-pup interactions by repeated maternal separation (MS) paradigm [4,5]. MS during the first postnatal days in rodents alters the normal programming of the hypothalamic-pituitary-adrenal (HPA) axis response to stress [5,6]. It has been reported that prolonged MS induces an enhanced neuroendocrine response to stress and it causes adverse effects in brain anatomy and function, synaptic plasticity, emotional responses and cognition in rodents [5,7–11]. Conversely, some studies reported that prolonged early MS in rodents might actually lead to enhanced resilience to stress during adulthood, expressed as better spatial learning and less anxiety-like behavior [5,12–14]. Enhanced maternal care and compensating neural adaptations could explain the contradictory results of different rodent models of early life stress induced by maternal separation [5,15].

In contrast, manipulations of rearing conditions involving exposure to complex housing conditions promoting social interactions, sensory-motor and cognitive stimulation with novel stimuli and physical exercise (environmental enrichment, EE) can counteract many possible detrimental effects of early stress by promoting neurogenesis, synaptic plasticity and modulating the HPA neuroendocrine response to stress [16]. However, there are also conflicting results regarding the behavioral effects of EE in rodents, with studies reporting decreased anxiety or depressive-like behavior, enhanced learning and memory, while others report increased anxiety, no significant effects on depressive-like behavior or learning and memory [17]. Strain differences and also different EE protocols and EE periods could be the cause of these discrepancies [17].

On the other hand, there is still limited information about the possible changes in functional brain networks underlying the possible beneficial effects of EE after exposure to early life stress. Energy production in eukaryotic cells takes place mainly in mitochondria, depending on a series of oxygen-dependent enzymes of the electron transport chain located in these organelles, and cytochrome c oxidase (CCO) is the key enzyme complex responsible for oxygen consumption in cells. The pivotal role of oxidative metabolism in mitochondria as a potential interface in neurodevelopmental programming after early life stress has been recently acknowledged [18–20]. Quantitative cytochrome c oxidase (CCO) histochemistry is a widely used method to evaluate sustained or long-term changes in brain regional oxidative metabolic capacity [21]. Previous studies using CCO histochemistry showed that MS induced changes in CCO activity and CCO connectivity in several rat brain regions [22,23]. However, CCO activity results differed across studies depending on the MS period chosen and rat strain used. In addition, studies using EE rearing in rats showed decreased CCO activity in brain regions involved in anxiety response in 3-month-old rats [24]. Conversely, another study using CCO histochemistry by the same authors reported that frontal and hippocampal networks showed more contribution than anxiety-related brain regions in 3-month-old rats after EE [25]. To our knowledge, the effects of EE after early stress induced by MS on regional brain energy metabolism have not been studied.

The aim of the current study was to verify the possible beneficial effects of EE after weaning in rats that were previously exposed to stress by MS during the three first weeks of life. For this purpose, animals were tested in a battery of behavioral tasks (anxiety-like behavior, depressive-like behavior, spatial learning and memory) and possible changes in functional regional brain connectivity and energy metabolism were also evaluated using CCO histochemistry.

## Materials and methods

### Animals

Litters from 20 pregnant Wistar rats from the central vivarium of the University of Oviedo (Spain) were cross-fostered to standardize litter size with 10–12 pups per dam (with similar sex ratio per litter). Animals were maintained at controlled room temperature (21±1°C) and humidity (65±5%) under a light-dark cycle of 12 h (lights on from 8:00 to 20:00 h). All experimental procedures were performed strictly following the European Union Directive 2010/63/UE on care and use of animals for scientific purposes and the Spanish legislation on care and use of animals for experimentation (Royal Decree 53/2013). In addition, all experimental procedures carried out in this study were approved by a local Ethics Committee of the University of Oviedo.

### Maternal separation procedure

Pups were randomly assigned to the following rearing conditions: (a) control litters were kept undisturbed except for weekly cage cleaning and weaning (Animal Facility Rearing or AFR); (b) MS litters were separated from their mothers 4 h daily from postnatal day (PND) 1 to PND 21 and litters were maintained together during this period in an incubator with controlled temperature (30°C) and 60±5% humidity (FIEM Cosmo Evo, Guanzate, Italy) following a previously published MS protocol [8,26–28].

### Environmental enrichment

Half of AFR or MS male pups assigned to EE rearing condition were independently housed in groups of 10 animals (AFR_EE and MS_EE groups) from PND 21 to 65 inclusive in a large cage (80 cm long x 75 cm wide x 86.5 cm high, FerPlast, Castelgomberto, Italy) [27]. The cage had numerous objects of different materials and shape (metallic and plastic balls, plastic toys, climbing ropes, plastic platforms and tubes) and a running wheel. Cage objects were rearranged and changed weekly as well as nesting materials. Feeding boxes and drinking bottles were changed weekly and placed at different locations in the enriched box.

**Fig 1:**
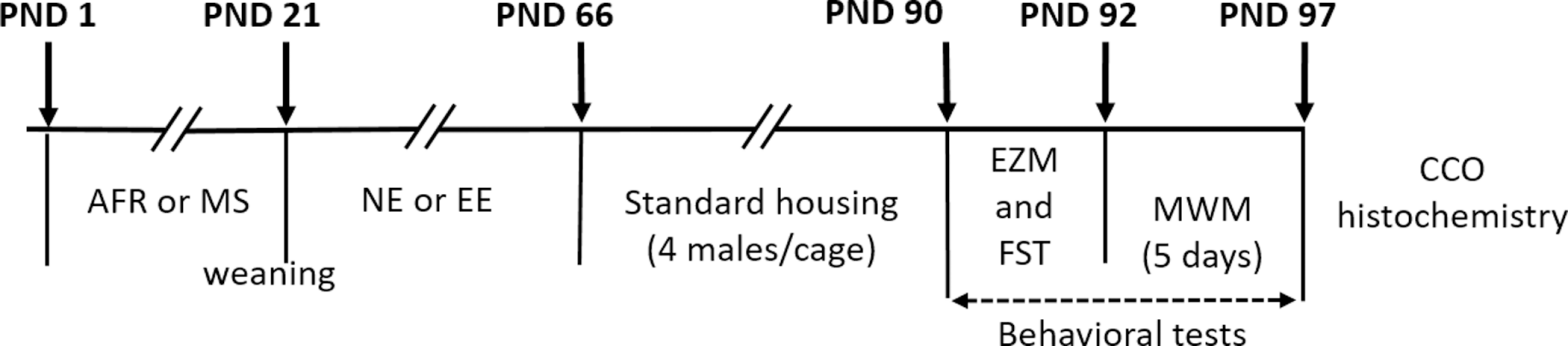
Experimental timeline and set-up. After birth, half of pups were randomly assigned either to daily maternal separation (MS, 4h/day) or standard animal facility rearing (AFR) until weaning at PND 21. Next, half of male animals were randomly assigned to environmental enrichment (EE) housing conditions or they were kept under standard housing conditions (non-ernriched, NE) from PND 21–66. Then, all rats were returned to their home cages under standard facility rearing (AFR) housing conditions. On PND 90, rats were tested in the elevated zero-maze (EZM) and the forced swim test (FST) on PND 90 and 91. From PND 92–97 rats were tested in the Morris water maze (MWM). All rats were sacrificed on PND 97 after MWM training, and brain tissue processed for CCO histochemistry.

The non-enriched AFR and MS male pups (AFR_NE and MS_NE groups) were maintained under standard AFR conditions in groups of 4 animals per standard cage (Eurostandard type IV 60 cm x 38 cm x 20 cm, Tecniplast, Italy) with food and water available ad libitum during the same period (PND 21 to 65). See Fig 1 for details about experimental set-up and timeline.

On PND 66, the enriched animals (AFR_EE and MS_EE groups) were returned to standard cages in groups of 4–5 animals until behavioral testing began in all experimental groups. This procedure is performed in order to favor equal conditions among experimental groups, to allow habituation and to optimize experimental manipulations until behavioral testing were performed, by allowing the enriched animals to habituate to the standard rearing conditions like the non-enriched groups during a few weeks [27,28].

### Behavioral procedures

Animals were handled during 4 days at least 30 min daily in order to habituate them to minimize stress response during experimental handling in the behavioral tasks. Rats from the four experimental groups (AFR_NE, MS_NE, AF, AFR_EE and MS_EE) were evaluated at PND 90 in a series of behavioral test following the same task sequence.

Forced swim test (FST). Depressive-like behavior was evaluated using the FST [29] during two additional consecutive days. The first day, rats were immersed in a glass cylinder (height: 50cm, diameter: 25 cm) filled with warm tap water (25±2°C) up to approximately 35 cm height. On the first test day, rats were allowed to swim during 15 minutes swim. Next day, animals were placed again in the cylinder during a 5-min session and the percent time spent floating immobile or swimming was measured using an automated video tracking system (Noldus Ethovision XT, Wageningen, The Netherlands).

Elevated zero-maze (EZM). Anxiety-like behavior was assessed using an EZM [30] the first day. Briefly, each animal was placed between an open and a closed sector, facing the inside of the closed sectorof the EZM, and then allowed to explore the closed and open sectors the maze for 5 minutes. Time spent exploring the open sectors was considered as reliable measure of anxiety-like behavior. In addition, we applied a correction for increased locomotor activity of time in open sectors by dividing the time in open sectors by the square root of the number of entries (Time by Entries index) [31]. The total distance moved was also calculated as a measure of locomotor activity and risk-assessment behavior was assessed by the total number of protected head-dips (when the rat stuck its head outside the maze border and below the maze floor from an enclosed maze sector). The rat behavior (in the EZM was measured by an automated video tracking system (Ethovision XT, Wageningen, The Netherlands). Protected head-dips were manually scored by an investigator, blind to the experimental group of each rat.

Morris water maze (MWM). Spatial learning and memory was assessed using the MWM as previously described [32]. Briefly, animals were trained during four days to find a hidden platform under the water using the visual cues available in the experimental room (spatial reference memory task). During the habituation trials the previous day, rats were trained to find a visible platform placed in the center of the water maze during four consecutive trials. During the next four training days, the platform was hidden under the water in the center of one virtual quadrant of the pool. Rats were allowed to swim in two daily 4-trial sessions with 1 h interval between them. In each trial, animals were released facing the walls from each water maze quadrant following a pseudorandom sequence during a maximum time of 60 s. It the rat failed to locate the platform, it was gently guided to it and allowed to be there during 15 s. Animals were dried with a towel and place in a plastic recipient during 15 s until the next trial. The total distance swum and the latency to find the hidden platform were measured using an automated video-tracking system (*Ethovision 3*.*1*, Noldus Information Technologies, Wageningen, The Netherlands).

The fourth day, rats were also evaluated in a spatial reference memory probe. In this task, the escape platform was removed immediately after the last trial and rats were allowed to swim during 60 s in the water maze. The total amount of time that the animal spent swimming in the quadrant that previously contained the platform (rewarded quadrant) was also measured. This variable is considered as an index of spatial reference memory unrelated to motor performance level [33].

### Cytochrome oxidase histochemistry

Ninety minutes after finishing the MWM task, 97-day-old rats were decapitated and their brains quickly removed. Brain tissue was frozen in isopentane (Sigma-Aldrich, Madrid, Spain) at -80°C and stored at -40°C. Serial 30-μm-thick coronal sections were cut with a cryostat microtome (Microm HM-505E, Heidelberg, Germany) and mounted on microscope slides. Brain sections were processed for quantitative cytochrome c oxidase (CCO) histochemistry as previously described [34–36] based on the original method by Gonzalez-Lima and Cada (1994) [21]. Briefly, sections were incubated during 1 h at 37°C in a solution containing 50 mg diaminobenzidine, 15 mg cytochrome c (Sigma, St. Louis, MO, USA) and 4 g sucrose per 100 ml phosphate buffer (pH 7.4; 0.1 M). The histochemical reaction was blocked by rinsing the slides with fixative solution containing buffered formalin for 30 min at 21°C, and sections were dehydrated and coverslipped. Brain homogenate standards of previously determined CCO activity were also included in each batch of slides to control for possible variations in staining intensity. CCO activity was assessed by optical densitometry of histochemical staining level using a high precision digital densitometry system (MCID Core, InterFocus Imaging Ltd., Linton, England). Briefly, relative optical density readings in the selected brain regions were normalized and converted to CCO activity units by measuring the optical density of the corresponding brain homogenate standard. A total of 12 measurements at three different anatomical levels of each brain region were used to obtain the mean CCO activity in each animal. The selected brain regions analyzed were: prelimbic (PL) area of the medial prefrontal cortex, the dorsal striatum (STR), the nucleus accumbens core (NAcC) and shell (NAcS) subdivisions, the hippocampal subfields of the dorsal (dCA1, dCA3, dDG) and ventral hippocampus (vCA1, vCA3). The selected brains regions were anatomically defined according to the atlas by Paxinos and Watson [37]

### Statistical analysis

Behavioral data were analyzed using SAS 9.14 PROC GLM (SAS Institute Inc., USA). In particular, effects of exposure to early stress (MS vs. AFR) and enrichment (EE vs. NE) in total immobility time in the FST, time spent in open arms, frequency of protected head-dips in the EZM, and time spent in escape quadrant during the spatial memory probe test in the MWM were analyzed by two-way ANOVAs and Benjamini-Hochberg’s *post-hoc* tests were applied in case of significant group differences (p<0.05). Benjamini-Hochberg’s (1988) *post-hoc* test as a powerful pairwise comparison procedure adjusting for false-discovery rates (FDR) in case of significant group differences [38].

The mixed-effects model repeated measures (MMRM) analysis with REML estimation (restricted maximum likelihood) estimation was applied for MWM escape latencies with stress (MS vs. AFR) and enrichment (EE vs. NE) conditions as independent factors, and training day as repeated-measures factor (see next paragraph for justification and further details about the selection of this particular approach of mixed model statistical analysis also for CCO activity data). Benjamini-Hochberg’s *post-hoc* tests were used for pair-wise comparisons in case of significant ANOVA results. Group differences in percent time spent in the rewarded quadrant during the memory probe task were evaluated using one-way ANOVA and Benjamini-Hochberg’s *post-hoc* tests were applied in case of significant group differences.

In order to analyze CCO activity data, we performed a randomized controlled trial (RCT) design in which the dependent variable was measured in nine brain regions using forty adult Wistar rats that were randomly assigned to the four experimental groups. Repeated measures data often involve measurements made on the same unit of analysis over time, although time is not a within-subject factor in this study. Likelihood-based mixed-effects models provide appropriate general analytic frameworks to determine whether fixed effects (i.e., the main effects and the effects associated with the brain regions by treatment interaction) and random effects (i.e., the effects associated with treatment for each rat) will be included in the model [39]. The mixed-effects model repeated measures (MMRM) analysis implemented herein included an unstructured modelling of regional brain activity and a within-subject error correlation structure. In this study, brain region is considered as a classification rather than a continuous variable. Following rejection of an omnibus hypothesis, we next determined the contrasts between the populations in which the means were not equal to zero. To control the family-wise error rate for all possible pairwise comparisons, the Hochberg (1988) step-up Bonferroni inequality [38] was applied using the ESTIMATE statement in SAS PROC MIXED and the HOC option in SAS PROC MULTTEST. The dataset was analyzed using MMRM with REML estimation as implemented in SAS Version 14.3 (SAS Institute, 2018) PROC MIXED.

In addition, in order to identify relationships (connectivity) between metabolic brain regions, a factor analysis was conducted using principal component analysis (PCA) extraction and varimax rotation available in SAS 9.14 PROC FACTOR SAS Institute Inc., USA). Prior to performing the analysis, the Kaiser-Meyer Olkin (KMO) measure of sampling adequacy and Bartlett’s test of sphericity (BTS) was used to examine the appropriateness of analysis. The factor score coefficients were also determined and used as variables for ANOVA.

## Results

### Behavioral data

**Fig 2:**
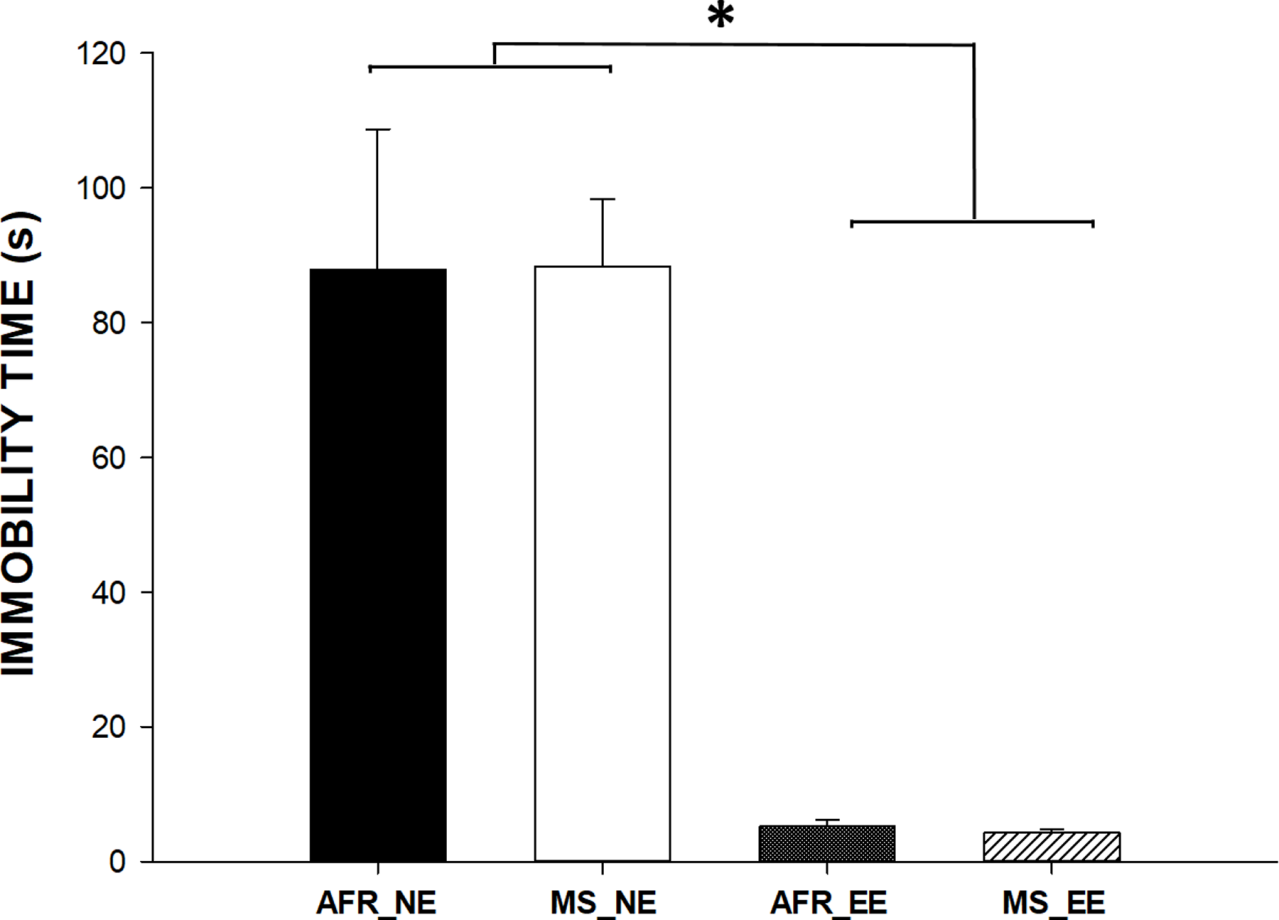
The groups with EE showed very low immobility time in the forced-swim test. *p < 0.001 vs EE groups, 2-way ANOVA.

The total amount of time spent immobile in the forced swim test (Fig 2) was significantly different between non-enriched (AFR_EE and MS_NE) and enriched groups (AFR_EE and MS_EE) [F(1, 36) = 26.21; *P*<0.001 See S1 Table to access original behavioral data.

**Fig 3:**
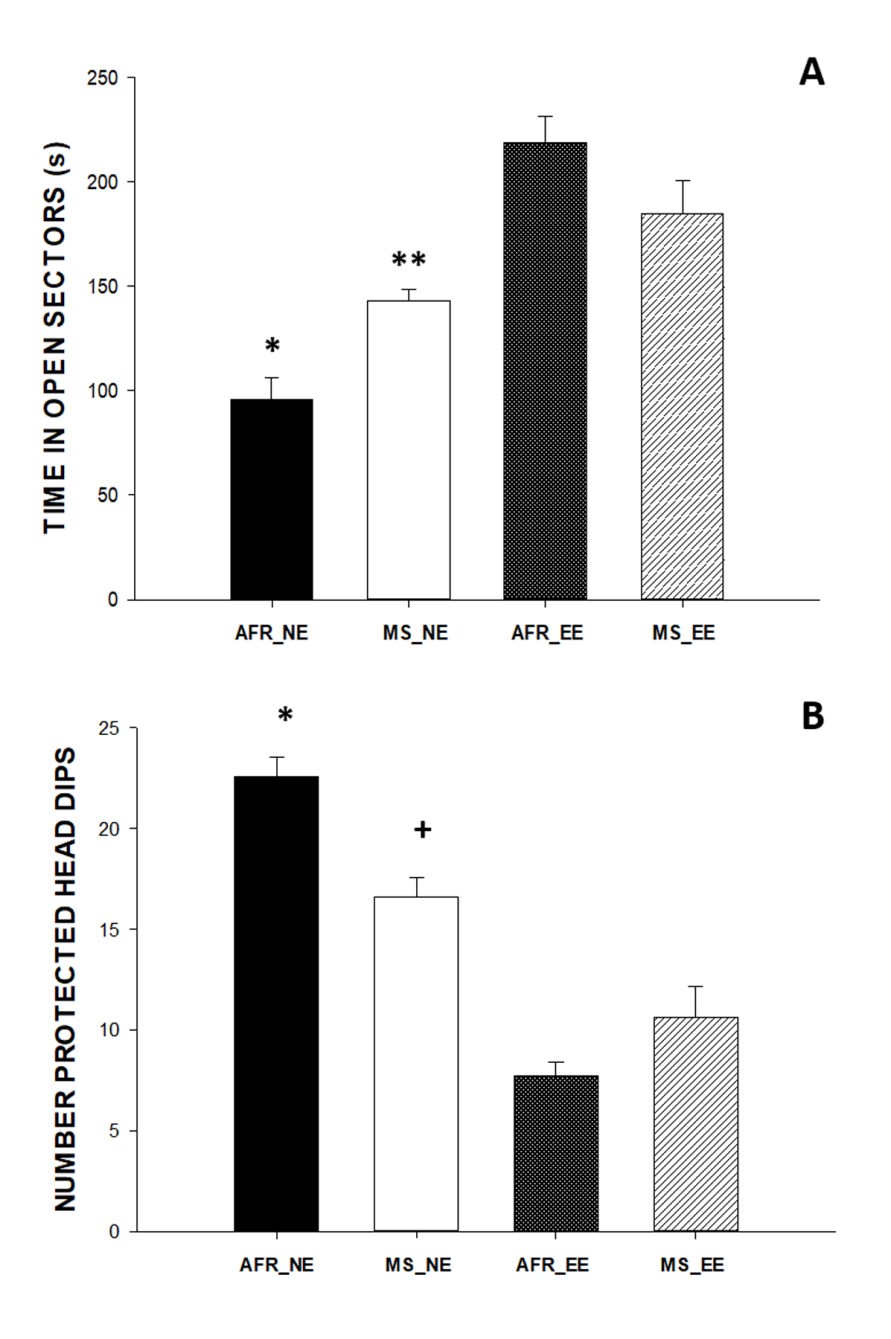
Results of anxiety-like behavior evaluated in the EZM. (A) Total time spent in open sectors during the 5-min trial was significantly lower in the non-enriched groups vs the EE groups. (B) Total frequency of protected head dips was significantly higher in the non-enriched groups, and the AFR_NE group showed the highest number of protected head dips. Each bar represents mean + standard error. *p < 0.01 vs other groups, **p < 0.001 vs AFR_EE, ^**+**^ p < 0.01 vs AFR_EE and MS_EE, Benjamini-Hochberg *post-hoc* tests.

As shown in Fig 3A, results of the EZM showed significant main effects of enrichment condition [*F*(1,36) = 49.1; P<0.001] and maternal stress x enrichment interaction in the time spent in open sectors of the EZM [F(1,36) = 11.96; *P*<0.001]. Hochberg’s post hoc tests showed that the time spent in open sectors was significantly lower in the AFR_NE group as compared with the rest of groups (Hochberg’s t tests; *P*<0.05]. In addition, the MS_NE group also spent less time exploring open sectors as compared with both the MS_EE group (*P* = 0.017) and the AFR_EE group (*P*<0.001). Similarly, the total number of protected head-dips (Fig 3B) also showed significant main effects of enrichment condition [F(1,36) = 90.31; *P*<0.001] and maternal stress x enrichment interaction [F(1,36) = 16.38; *P*<0.001]. Post-hoc analysis revealed that the number of protected head-dips was significantly higher in the AFR_NE group versus the rest of groups (*P*<0.0001). The MS_NE group also showed significantly more protected head-dips as compared with both the AFR_EE and MS_EE groups (*P*<0.01). There were no significant differences in the total distance moved.

Results of the mean escape latencies of the spatial learning task in the MWM (Fig 3A) revealed significant main effects of enrichment condition [F(1,37) = 31.47; P<0.001], training day [F(3,114) = 43.16; P<0.001] and interaction between enrichment conditions and training days [F(3,114) = 3.01, *P* < 0.05]. Hochberg’s post-hoc analysis of the interaction showed that the non-enriched groups had significantly greater escape latencies as compared with the EE groups on days 1, 2,and 3 (*P*<0.0001). However, no significant differences between non-enriched and EE groups were found on day 4 (*P*<0.085).

**Fig 4:**
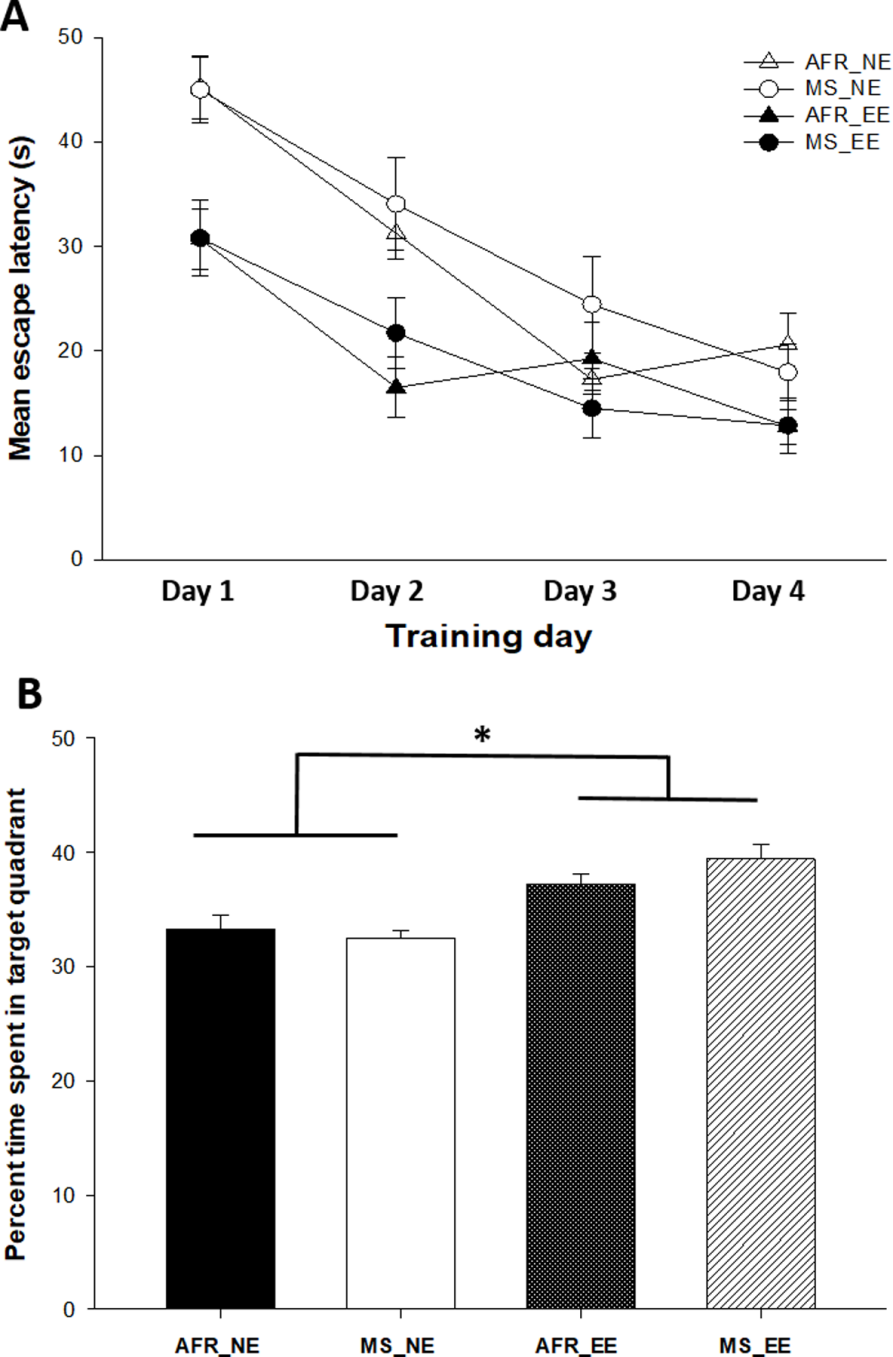
Evaluation of spatial learning and memory in the Morris water maze. (A) Mean escape latency was significantly lower in the groups with Environmental enrichment (EE) on days 1 and 2. (B) The percent time spent in the pool quadrant where the platform had previously been placed in the probe trial (spatial memory) was also significantly higher in the EE groups. *p < 0.05 vs environmental enrichment (EE) groups, Benjamini-Hochberg *post-hoc* tests. See results section for details.

There were significant main effects of enrichment condition in the MWM spatial memory probe test [F(1,36) = 26.21; *P*<0.001]. All groups reached the learning criterion (more than 25% time spent in the target quadrant) (Fig 4B).

### CCO activity

#### The mixed-effects model repeated measures (MMRM) analysis

**Fig 5:**
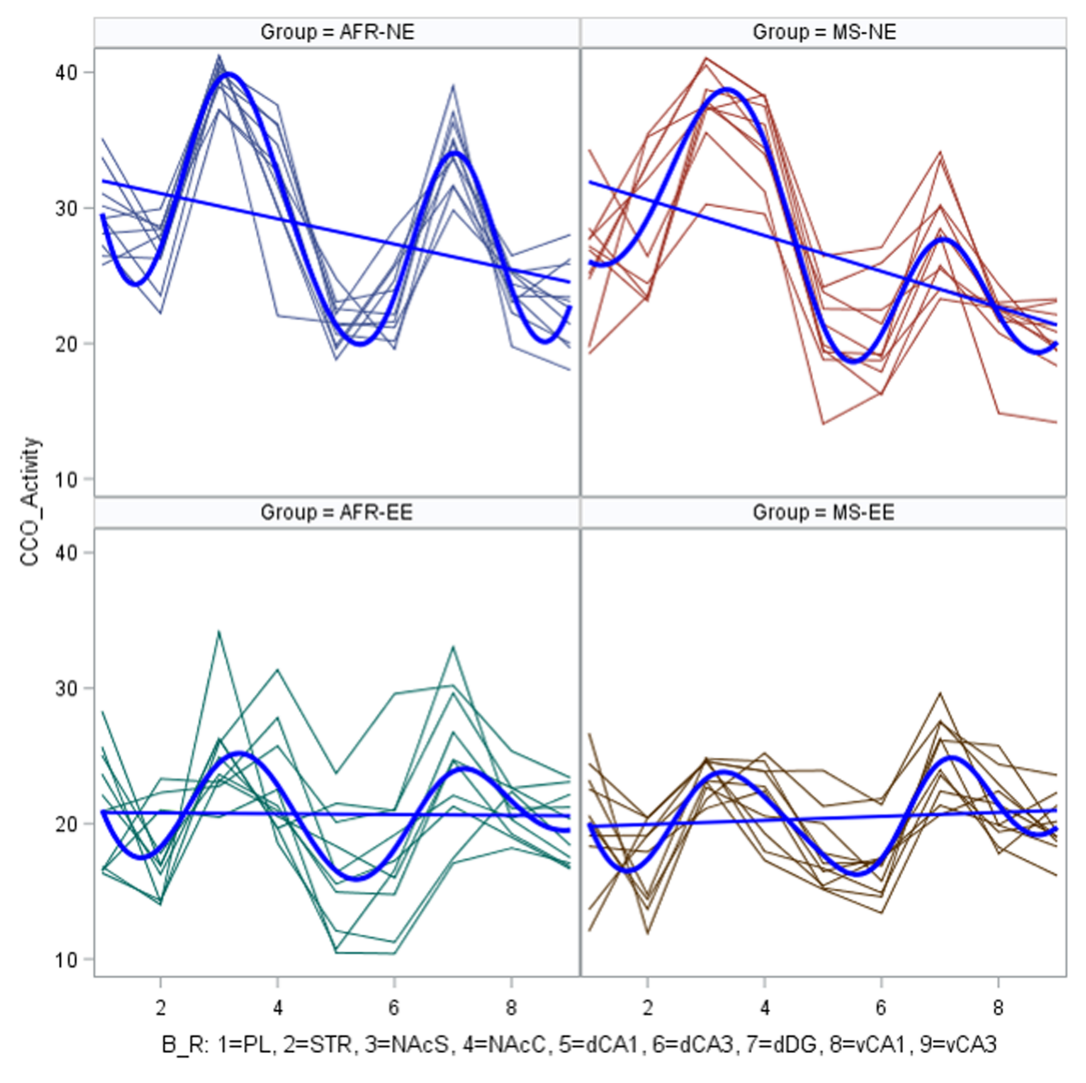
Spaghetti plot of CCO activity data for each animal in the brain regions of interest. Mean value (solid line) of the different experimental groups.

The effects of treatment on the metabolic brain regions within animal are shown in Fig 5. This plot indicates that CCO activity is consistently higher in the non-enriched groups (AFR_NE and MS_NE) than in the enriched groups (AFR_EE and MS_EE) across all regions. The characteristics of these data suggest that fixed effect and random effects are likely to be significant in a mixed model. See S2 Table to access original CCO data.

In the absence of a theory providing contrasting data, we used a data-driven strategy to move toward a simpler structure by eliminating predictors or (co)variances that did not appear to be related to the dependent variable. Based on this modeling strategy, the selected model includes fixed effects of the region by treatment interaction and random intercepts. There were significant effects of experimental groups [F(3,36) = 49.50, *P*< .0001], brain regions [F(8,882) = 78.40, *P*< .0001] and interaction between group and brain region [F(24,288) = 8.47, *P*< .0001]. Results also indicate that there were statistically significant differences in CCO activity between animals (Z = 2.88; *P* = .002) and within animals (Z = 12.01; *P*< .0001).

Hochberg’s post hoc tests revealed significant differences between AFR_NE and MS_EE groups [t(36) = 9.69; *P* < .0001], AFR_NE and AFR_EE groups [t(36) = 9.30; *P* < .0001], MS_NE and MS_EE groups [t(36) = 7.69; *P* < .0001], and MS_NE and AFR_EE groups [t(36) = 7.31; *P* < .0001].

The next step was to examine whether the CCO activity change was different for the non-enriched *versus* EE groups across different brain regions. Linear combinations of means are estimated and compared for this purpose using the LSMEANS statement of the PROC MIXED. The least-squares means are estimates of the two groups evaluated in the brain regions of interest. As shown in Table 1, mean CCO activity of EE and non-enriched groups are significantly different for most brain regions analyzed, after applying Hochberg’s sequentially rejective Bonferroni procedure. See S3 Table for detailed statistical analysis and post-hoc tests of the mixed-effects model repeated measures analysis of CCO data.

**Table 1:** Results of the statistical analysis of CCO activity (n = 10 per region and group) of the four experimental groups.

	AFR_NE			MS_NE			AFR_EE			MS_EE		
**PL**	29.62	±	0.96	26.10	±	1.38	22.18	±	2.14*	20.07	±	1.45*
**STR**	26.77	±	0.91	28.55	±	1.61	17.80	±	1.11*	16.86	±	1.00*
**NAcS**	39.45	±	1.34	37.37	±	1.29	24.93	±	1.36*	23.40	±	0.77*
**NAcC**	32.67	±	1.55	34.92	±	0.94	23.16	±	1.28*	21.54	±	0.89*
**dCA1**	21.43	±	0.57	21.00	±	1.10	16.37	±	1.44*	17.91	±	0.93*
**dCA3**	23.32	±	0.90	20.44	±	1.19	17.70	±	1.74*	17.07	±	0.87*
**dDG**	34.27	±	0.88	28.29	±	1.18	24.68	±	1.69*	24.90	±	0.94*
**vCA1**	23.89	±	0.61	21.70	±	0.81	21.12	±	0.68	21.20	±	0.83
**vCA3**	22.83	±	1.01	20.21	±	0.85	19.66	±	0.85	19.79	±	0.68

Data are expressed as mean± standard error.

* *P* < .0001 EE vs. NE condition (Hochberg’s *post-hoc* tests).

#### Principal component analysis

The changes in CCO activity induced by both non-enriched and enriched treatment groups were also studied using a variable-reduction procedure similar to factor analysis (i.e., principal component analysis). The null hypothesis that the correlation matrix was an identity matrix was rejected by BTS for every factor analysis [χ^2^(36) = 62.35, *P* = .0020; χ^2^(36) = 91.24, *P* < .0001], therefore there is a statistically significant interrelationship between brain regions. The KMO measure of sampling adequacy yielded a value of 0.56 and 0.52, respectively, for both non-enriched and EE groups.

The results shown in Table 2 (left panel) indicate that in the non-enriched animals (AFR_NE and MS_NE groups), the NAcS, dCA3 and dDG brain regions loaded highest on the component 1, the STR, vCA1 and vCA3 brain regions loaded highest on the component 2, and the PL, NAcC and dCA1 brain regions loaded highest on the component 3. No significant group differences were found for any of these components in subsequent analyses using component score as dependent variables [(F(1,18) = 1.19, *P* = .2897; F(1,18) = 1.92, *P* = .1829; F(1,18) = 0.17, *P* = .6833].

**Table 2:** Loadings for principal components using varimax rotation and percent of variance accounted for by components.

	Non-enriched groups			Enriched groups		
	Principal component analysis (components)			Principal component analysis (components)		
Brain region	1	2	3	1	2	3
PL	.2839	.1449	-.**7814**	.**8443**	-.1073	-.2961
STR	.2052	-.**8091**	.2358	-.1964	.0323	.**8694**
NAcS	.**8087**	-.2437	.1168	-.0805	.0156	-.**5586**
NAcC	.2001	-.1485	.**7233**	.3154	.**5527**	.**5859**
dCA1	.4958	.2771	.**6589**	.**8232**	.3402	.1131
dCA3	.**7922**	.2146	.0823	.**6785**	**.5261**	.2527
dDG	.**8566**	.1509	-.0524	.**8878**	.2674	.0558
vCA1	.2033	.**8570**	-.1321	.1272	.**7766**	-.4911
vCA3	.1673	.**8834**	.1332	.1889	.**7937**	.1499
Variance explained	32%	26%	15%	41%	21%	12%

Note: Loadings in bold are values above 0.50.

In the enriched animals (right panel; AFR_EE and MS_EE groups), the PL, dCA1, DCA3, and dDG brain regions loaded highest on the component 1, the NAcC, dCA3, vCA1 and vCA3 brain regions loaded highest on the component 2, and the STR, NAcS and NAcC brain regions loaded highest on the component 3. Again, no significant group differences were found in any of these components in subsequent analyses using component score as dependent variables [(F(1,18) = 0.11, *P* = .7553; F(1,18) = 0.01, *P* = .9062; F(1,18) = 1.68, *P* = .2116]. It should be mentioned that some of these regions (i.e., NAcC and dCA3) loaded moderately in more than one component, suggesting that these regions were part of both circuits and may have interacted with each other.

## Discussion

Globally, no behavioral effects of prolonged MS were observed in the tests applied, except for anxiety-like behavior. MS_NE rats spent more time in open arms as compared to the AFR_NE group, suggesting decreased anxiety-like response. In addition, MS_NE animals showed less risk assessment behaviors as shown by decreased frequency of protected head-dips. Therefore, the behavioral pattern of MS_NE animals seems to be compatible with decreased anxiety-like behavior in contrast with previously reported results by other authors [23].

Indeed, the efficacy of the EZM or EPM in screening anxiety modifying interventions has increased with the use of ethological measures such as head-dipping and protected stretches [40–42]. Accordingly, it has been reported decreased anxiety-like behavior after MS in from PND1-21 in young Wistar rats [43].

Prolonged MS during the entire lactation period (PND 1–21) may not lead to abnormal development of the stress response mediated by the HPA axis and associated behavioral outcomes [12,23,44]. In particular, early MS during the first two weeks of life (PND 2–15) seems to cause increased anxiety-like behavior and depressive-like behavior [44]. Early MS from PND 2–15 overlaps most of the stress hypo-responsive period (PND 2–14), but if MS is prolonged beyond this period (PND 2–21) the HPA axis is already mature, and it could match the environmental stressors [5,45]. In fact, blunted corticosterone response after restraint stress and no anxiety-like response in elevated plus-maze have been reported after prolonged MS from PND 1–21 in male Wistar rats [46]. In addition, no differences in anxiety-like behavior after MS in adult male Wistar rats have been reported in other studies [47,48]. The discrepancies in results regarding anxiety-like behavior after MS could be explained by different factors like differences in MS protocols and rat strain, age. It is possible that prolonged MS resulted in increased maternal care that suppresses or decreases HPA-reactivity of pups [49] as well as anxiety-like behavior [50]. Moreover, risk assessment behavior is considered a more sensitive index of emotional reactivity [51,52]. Our results show decreased risk assessment behavior in MS_NE animals as compared with AFR_NE rats, as previously described by other authors [46]. There is a growing awareness that risk assessment behaviors provide a more complete profile of anxiety-related behaviors and may be a more sensitive index of emotional-reactivity [51–53].

Our results support the ‘stress match-mismatch’ hypothesis, stating that exposure to early life stress may trigger adaptive programming responses, thereby rendering an individual to be better adapted to aversive or stressful situations later in life [4,5,54]. In humans, clinical evidence indicates that early life adversity does not necessarily lead to the development of mental disorders in adulthood like depression and anxiety by most people [55]. In fact, many individuals might learn to cope with moderate stressful events by experiencing early life stress, as the so-called stress match-mismatch hypothesis suggests [54,56]

In agreement with the stress mismatch hypothesis, no differences in depressive-like behavior in the FST and spatial learning and memory in the MWM were found between MS_NE and AFR_NE groups. These results agree with previous studies by several authors using the same or different MS protocols and rat strains [12,23,57–60]. Probably, timing and duration of early life stress used in different MS protocols could be the critical factor responsible for the apparently contradictory behavioral results of many studies, as it has been recently suggested [61].

In this regard, it has been previously reported increased or no effects on anxiety-like behavior in adult male rats using slightly different early MS periods (PND 2–15 or PND 1–10) [44,57]. Moreover, detrimental or no adverse effects on spatial learning and memory have been reported after comparison of different MS periods (PND 1–10 vs. PND 1–21 or PND 2–9 vs. PND 14–21) [57,58]. However, studies greatly differ in the MS periods chosen, in particular the stress hypo-responsive period in rats. Therefore, additional studies should be performed to compare in adult rats the behavioral effects of early (PND 2–15) versus complete (PND 2–21) MS periods.

On the other hand, exposure to EE had substantial effects on the behavioral tests applied. In particular, it apparently decreased anxiety-like and depressive-like behaviors in both MS_EE and AFR_EE animals as compared with MS_NE and AFR_NE respectively. In this regard, it has been reported that EE could revert the effects of MS on stress reactivity [16]. In addition, EE could also improve spatial memory in rats and decrease anxiety-like and depressive-like behaviors as reported by other authors using a similar EE protocol and rat strain [24,62]. However, after careful examination of individual behavioral results (see behavioral data in S1 Table) rats of the EE groups spent an exceedingly high amount of time in open sectors of the EZM (average 70% of total time). Moreover, several animals in both AFR_EE and MS_EE groups spent almost the entire testing time into open sectors. Therefore, the behavior of EE animals could be better considered as non-adaptive, and even as anxiety-like behavior. EE protocols and behavioral effects greatly differ among researchers [17]. In our case EE did not cause clear beneficial effects on emotional responses in both MS and AFR rats. In this regard, EE has been associated with decreased emotionality in novel situations and lower HPA reactivity [63,64], as well as enhanced brain plasticity and hippocampal neurogenesis [17]. Conversely, EE in groups of 10 rats has been associated with increased plasma corticosterone concentrations and larger adrenal glands, a result suggesting chronic stress conditions [63]. Some authors have proposed that the benefits of EE on anxiety-like behavior are not clear, and proposed the ‘stress inoculation’ hypothesis of EE as a form of chronic mild stress [65]. Moreover, EE has also been reported to increase anxiety-like behavior in rodents using different tests unrelated with locomotor activity to evaluate anxiety [66]. In addition, immobility time in the FST was also too low in EE animals (below 1% of total time) a result suggesting increased anxiety or impulsiveness reported after exposure to chronic stress in Wistar rats or 5-HT depletion [67,68]. In fact, the original interpretation of immobility in the FST initially regarded as ‘behavioral despair’ or ‘learned helplessness’ related with depression-like behavior [69] is not always straightforward. Another interpretation of immobility in the FST is that it would be an adaptive response to stressful situations [70,71] or a measure of impulsive behavior [68]. In our case, the almost absence of immobility in the FST in EE rats could be probably interpreted as a non-adaptive response to stress, manifested as anxiety-like behavior, or high impulsivity. Therefore, EE conditions used here seem to induce chronic stress in the animals, as shown by the abnormal behavioral response in both the EPM and the FST. However, spatial learning and memory tested in the MWM was not negatively affected by EE. In fact, percent time spent in target quadrant of the MWM (a test of spatial memory ability) was significantly higher in EE groups, apparently showing significantly better spatial memory as compared with NE groups. In this regard, it has been reported that high stress levels are deleterious for spatial learning, but there is a linear facilitating effect of stress on spatial memory consolidation [72].However, it should not be discarded that the AFR conditions applied here (rearing animals in groups of 4 per cage) could be also considered as mild social EE conditions, especially compared with studies using single- or double-housing conditions.

Prolonged single-housing in rats (not mice) is considered as a social isolation stress model that causes activation HPA axis, and it has been used to model signs and symptoms of mental disorders and neurological conditions such as anxiety, depression, schizophrenia, epilepsy and memory loss [73]. In particular, our research group has recently reported anxiety-like behavior, impaired working memory, and altered brain CCO activity in adult rats after 12 weeks of single-housing, as compared with rats reared in groups of 4 [74].

It has been demonstrated that housing male rats in groups of 4 causes less stress (measured by cardiovascular responses and cage behavior) as compared to housing them in groups of 2 [75]. We used the standard facility housing conditions recommended by our ethics committee, housing adult rats in groups of 4 in European Eurostandard type IV cages, allowing up to 6 rats of 450 g maximum. Conversely, it has been reported that when rats are housed with an approximately equivalent floor area per animal, those housed with more conspecifics experience greater levels of social stressors than those housed with a single cage-mate [76]. However, these authors were unable to confirm the hypothesis that increased housing density of animals or a decreased space allocation would result in increased numbers of anxiety-like behaviors. Therefore, in our case, apparently greater levels of exposure to social stress could be associated with EE conditions (groups of 10 rats in a large cage) as compared to standard facility rearing conditions (4 rats in a smaller cage). s. As regards to the role of ‘social stimulation’ (number of subjects per cage) component of EE, it has been reported that its beneficial effects on brain plasticity and cognition are minor as compared to physical stimulation (sensory stimulation and physical exercise) associated with EE in Wistar rats [77]. Therefore, we do not believe that the AFR housing conditions used here could be considered as conventional or ‘mild’ EE conditions.

Moreover, analysis of CCO activity showed that EE groups had lower metabolic capacity in most brain regions measured, but MS did not affect CCO activity in both AFR and EE groups. Decreased brain CCO activity after EE has been previously reported by several authors in rats and mice [24,78]. Decreased brain metabolic capacity after EE may be related with higher metabolic efficiency requiring less energy demands by brain cells to perform behavioral tasks. In fact, EE groups performed better the spatial learning task in the MWM as compared to the NE groups. EE groups had lower escape latencies in the MWM spatial learning task, as well as better spatial memory recall as shown by greater time swimming in the escape quadrant of the MWM. Conversely, decreased brain CCO activity or inhibition of mitochondrial complexes related with brain energy metabolism have been found after exposure to chronic stress in rodents [79–83]. In addition, lower CCO activity in the prefrontal cortex of patients with major depression [84] as well as decreased energy metabolism in several limbic brain regions [85]. It is known that the respiratory chain function (including CCO or complex IV) in mitochondria can be disrupted by neurochemical mediators of anxiety and stress like catecholamines and glucocorticoids [19]. Significant CCO activity decreases were found in the prelimbic cortex, striatum, nucleus accumbens, and the dorsal hippocampus in both EE groups. In particular, chronic social defeat stress in rats is also associated with decreased CCO in the anterior cingulate cortex, the nucleus accumbens, and the hippocampus (CA1, CA3, and dentate gyrus) [79,86,87].

In addition, decreased glucose uptake measured by PET in the striatum has been reported in a depression model in rats after chronic corticosterone administration [88]. Decreased CCO activity in brain regions included in the mesolimbic dopamine pathway play a key role in the motivational and/or hedonic processes is associated with anxiety and depression [89,90]. The nucleus accumbens is particularly relevant in motivated behavior and it has been involved in the regulation of anxiety and depression [91]. In this regard, high anxiety in rats under social stress is associated with lower mitochondrial function and reduced ATP formation in the nucleus accumbens [87,90]. Probably, abnormal anxiety-like behavior in the EZM and impulsive- or anxiety-like behavior in the FST would be associated with decreased CCO activity in limbic regions induced by chronic stress after EE rearing.

However, our CCO results do not agree with a previous study reporting increased CCO activity in most brain regions after EE in 3-month-old Wistar rats [25]. The discrepancy with our results may be due to differences in the spatial memory task used (radial water maze versus MWM) and most importantly the age of animals when the EE protocol was applied (3-month-old rats versus 21-day-old rats in our case). Differences in spatial orientation strategies and brain networks used by adult and aged rats during the spatial learning task could be an additional relevant factor to explain this discrepancy. Moreover, the effects of EE on brain development and neural plasticity are clearly age-dependent [92]. In this regard, EE during the early postnatal period around weaning as reported here can modify neuronal differentiation and affect neurodevelopmental programming of stress neuroendocrine response, as well as hippocampal and prefrontal cortex volumes and morphology [7,26,92]. The lack of effect of MS on brain CCO activity is in agreement with previous results of our research group using the same prolonged MS period from PND 1–21 [23]. In addition, CCO results matched behavioral outcomes, since no overall significant differences were observed between AFR and MS groups.

On the other hand, EE increased variability of mean CCO activity in AFR rats. However, this individual variability was not clearly observed in behavioral data (except for immobility time measured in FST of AFR rats). We do not have a straightforward explanation for this individual variability in regional brain metabolism, but it is interesting to note that the effects of EE are complex and dependent on individual differences in brain metabolism of AFR animals. It is possible that individual exposure to different levels of social stress (dominant vs. subordinated animals) involved in the EE paradigm [76] would explain this variability in brain metabolism. However, MS animals seem to have more uniform response to EE regarding brain metabolism, that it might be possibly related with their less anxious emotional profile as shown by EPM test.

Principal component analysis of CCO activity showed different brain regions loading on three different components for both enriched and non-enriched groups. In particular, enriched groups slightly differed in the brain regions loading for component 1 (prelimbic region), component 2 (nucleus accumbens core and dorsal CA3 hippocampal region) and component 3 (striatum, nucleus accumbens shell). Brain regions loading for component 1 are related with spatial memory and goal-directed or rewarded behavior (dorsal hippocampus and nucleus accumbens shell), regions loading for component 2 seem to be related with motor and emotional behavior (striatum, ventral hippocampus) and component 3 regions are also related with motivation and goal-directed behavior (striatum, nucleus accumbens). PCA is a data-driven multivariate statistical technique that simplifies or reduces the dimensions of measured variables (here CCO activity in different brain regions) to find a new set of variables (principal components) that can be understood as hidden patterns of temporal cross-correlations or co-variances between measured variables. PCA has been applied to determine functional brain connectivity of brain regions in rodents using CCO histochemistry [25,82] and functional neuroimaging studies [93]. Component loading indicates the extent to which each brain region is related to each component. High component loading indicates a relevant contribution of a particular brain region for each component. Thus, components could be interpreted as brain networks involved in particular brain functions and psychological constructs (emotion, cognition, etc.). Brain regions showing high loadings (higher than 0.5) on a particular component (explaining most of the data variance) could be considered as part of the same functional brain network. Although both the non-enriched and enriched groups showed common functional brain networks related with the spatial memory task, the enriched groups recruited more brain regions in the three different components found in the PCA analysis. Probably, the additional brain regions recruited by the enriched groups would be partly related with their better spatial memory performance in the MWM task.

In particular, the contribution of the PL cortex and the dCA1 hippocampal region for component 1 is clearly related with spatial memory acquisition and consolidation, as we have previously reported [94–96]. Moreover, STR and NAcS involvement in component 3 of enriched groups may be linked to their higher locomotor activity and less immobility in the FST, and the MWM. PCA results show that EE increases functional brain connectivity as compared to NE condition, as shown by the higher number of brain regions recruited after PCA of CCO activity for each component. Our results agree with several studies reporting complex large-scale brain networks involving the dorsal hippocampus and the PL cortex in a particular spatial memory tasks after EE in rats [25,62].

However, differences between enriched and non-enriched groups in brain regions mainly contributing to the different principal components would be also associated with the effects of chronic stress on brain metabolism. In this regard, limbic regions associated with anxiety-like behavior and stress response significantly differed between enriched and non-enriched groups, particularly in components 2 and 3, like the nucleus accumbens shell, the striatum, the dorsal hippocampus, and the prelimbic cortex. As mentioned earlier, these brain regions also showed decreased CCO activity in the EE groups, as related with increased anxiety-like behavior [79,90]. Accordingly, differences in functional brain connectivity between enriched and non-enriched groups in the above-mentioned limbic regions would underlie the effects of chronic stress on brain networks associated with anxiety and stress response, as reported by other authors [79,81,82,87].

In sum, exposure to early life stress could lead to resilience to stress in adulthood depending on timing of MS. In addition, EE during the early postnatal period seems to alter anxiety-like behavior and/or depression-like behavior, but beneficial effects on spatial memory, even after exposure to early life stress by maternal separation. Moreover, EE could alter the programming of functional neurodevelopment during particular time windows, resulting in differential pattern of functional brain networks related with particular aspects of cognition and emotion. In view of the results found, we should consider that ‘environmental enrichment’ conditions should be standardized and adopted as an standard rearing condition for experimental animals, as recommended by the current European Directive 2010/63/EU on the care and use of laboratory animals. However, this directive does not determine specific enrichment conditions by simply stating that ‘environmental enrichment in animal enclosures shall be adapted to the species and individual needs of the animals concerned’ (section 3.3 of Directive 2010/63/EU) [97]. Mandatory environmental enrichment conditions still need to be evaluated for their standardization in rodents, as already reported by other authors [98]. Consequences of EE could be detrimental or beneficial for laboratory animals, depending on the particular housing conditions and the animal species and strain [99]. Future studies should provide additional knowledge of the specific housing conditions effects on brain and behavior in order to standardize EE protocols, and to establish appropriate and ethical regulations for rearing laboratory animals.

## Supporting information

S1 Table Original behavioral data. (XLSX)

S2 Table CCO activity data. (DOCX)

S3 Table Detailed statistical analysis of CCO activity results. (DOCX)
