# Correction: Environmental enrichment effects after early stress on behavior and functional brain networks in adult rats

**DOI:** 10.1371/journal.pone.0231586

**Published:** 2020-04-02

**Authors:** The PLOS ONE Staff

## Notice of republication

An incorrect version of S1 Table was published in error. The publisher apologizes for the error. This article was republished on March 18, 2020, to correct for this error. Please download this article again to view the correct version.
